# Supplementary material for: Prevalence of family-based elder abuse and its associated factors in Gandaki Province of Western Nepal: A cross-sectional study
Source: PLoS One. 2025 May 14;20(5):e0323713. doi: 10.1371/journal.pone.0323713 (PMC12077681; doi:10.1371/journal.pone.0323713)
Supplement: S1 Tables — (DOCX) [file pone.0323713.s001.docx]

**S1 Table. Demographic and socioeconomic characteristics of study districts.**

| **Attributes** | **National** | **Gandaki Province** | **Study districts** | | |
| --- | --- | --- | --- | --- | --- |
|  |  |  | **Manang** | **Tanahu** | **Nawalpur** |
| Total number of households | 6,666,937 | 662,480 | 1,572 | 88,583 | 93,925 |
| Total population | 29,164,578 | 2,466,427 | 5,658 | 321,153 | 378,079 |
| Total 60+ population | 2,977,318 | 329,107 | 764 | 42,702 | 40,065 |
| Average household size (persons per household) | 4.37 | 3.72 | 3.60 | 3.63 | 4.03 |
| Population density (no. of people per square kilometer of area) | 198 | 115 | 3 | 208 | 265 |
| Annual average population growth rate (in percentage) | 0.92 | 0.25 | -1.39 | -0.06 | 1.86 |
| Sex ratio (per 100 females) | 95.59 | 90.37 | 129.44 | 87.74 | 88.86 |
| Old-age sex ratio (per 100 females) | 94.16 | 87.26 | 78.50 | 86.44 | 91.65 |
| Old-age dependency ratio (in percentage) | 16.48 | 21.25 | 18.87 | 21.22 | 16.51 |
| Aging index (in percentage) | 24.91 | 23.12 | 64.42 | 37.74 | 28.59 |
| *Note*. Old-age sex ratio is the ratio of males to females of age 60 years and above. The old-age dependency ratio is between the population aged 60 and above and those aged between 15 and 59. The aging index is the ratio of the population aged 65 and above to those aged 0-14. | | | | | |

*Source*: National Population and Housing Census 2021, Nepal.

**S2 Table. Sampling technique.**

| **Strata** | **Size /Population*** | **Proportion** | **Proportionate**  **allocation** | **Equal**  **allocation** | **Proportionate**  **cluster** | **Required**  **cluster** | **Sample**  **size** |
| --- | --- | --- | --- | --- | --- | --- | --- |
| **1** | **2** | **3=(2/N)** | **4=(3*N^S^)** | **5** | **6=(4/5)** | **7** | **8=(7*5)** |
| Tarai Urban | 24,166 | 0.44 | 273.66 | 22 | 12.44 | 12 | 264 |
| Tarai Rural | 8,122 | 0.15 | 91.98 | 22 | 4.18 | 4 | 88 |
| Hill Urban | 8,022 | 0.15 | 90.84 | 22 | 4.13 | 4 | 88 |
| Hill Rural | 13,407 | 0.25 | 151.83 | 22 | 6.90 | 7 | 154 |
| Mountain Rural | 679 | 0.01 | 7.69 | 22 | 0.35 | 1 | 22 |
| **Total** | **54,396** | **1.00** | **616.00** |  | **28.00** | **28** | **616** |

*Note.* *Size reported based on National Population and Housing Census 2011, Nepal; N=Total population size=54,396; N^S^ =Total sample size = 616.

**S3 Table. Operational definition of overall abuse and its sub-types.**

| Overall Elder Abuse | The presence of one or more of the following forms of abuse by family members. |
| --- | --- |
| Physical Abuse | Any form of physical harm by family members, such as beating, pushing, or causing bruises by gripping arms tightly. |
| Psychological Abuse | Any form of verbal or mental abuse by family members, such as the use of abusive language, insults, threats for minor mistakes, abandonment in unfamiliar places, or isolation from social interactions. |
| Caregiver Neglect | Acts of neglect by informal caregiver(s) in the family, resulting in older adults feeling isolated despite living with family, lack of communication and unresponsiveness to older adults, delayed meal delivery, disregard for health, or family members not searching for them when they are outside for extended periods. |
| Financial Abuse | Abuse related to finances by family members, such as unauthorized transfer of properties or belongings, theft of cash or valuables, or coercion in the transfer of possessions. |
| ^1^Legal Abuse | Abuse of identification or lack of essential legal documents such as citizenship and voter cards, prolonged court cases, or police refusal to register complaints from older people. |
| Sexual Abuse | Any accusations of sexual assault by family members. |

*Note:* ^1^Excluded in this study.
