# Supplementary figures and images for: Prevalence of family-based elder abuse and its associated factors in Gandaki Province of Western Nepal: A cross-sectional study
Source: PLoS One. 2025 May 14;20(5):e0323713. doi: 10.1371/journal.pone.0323713 (PMC12077681; doi:10.1371/journal.pone.0323713)

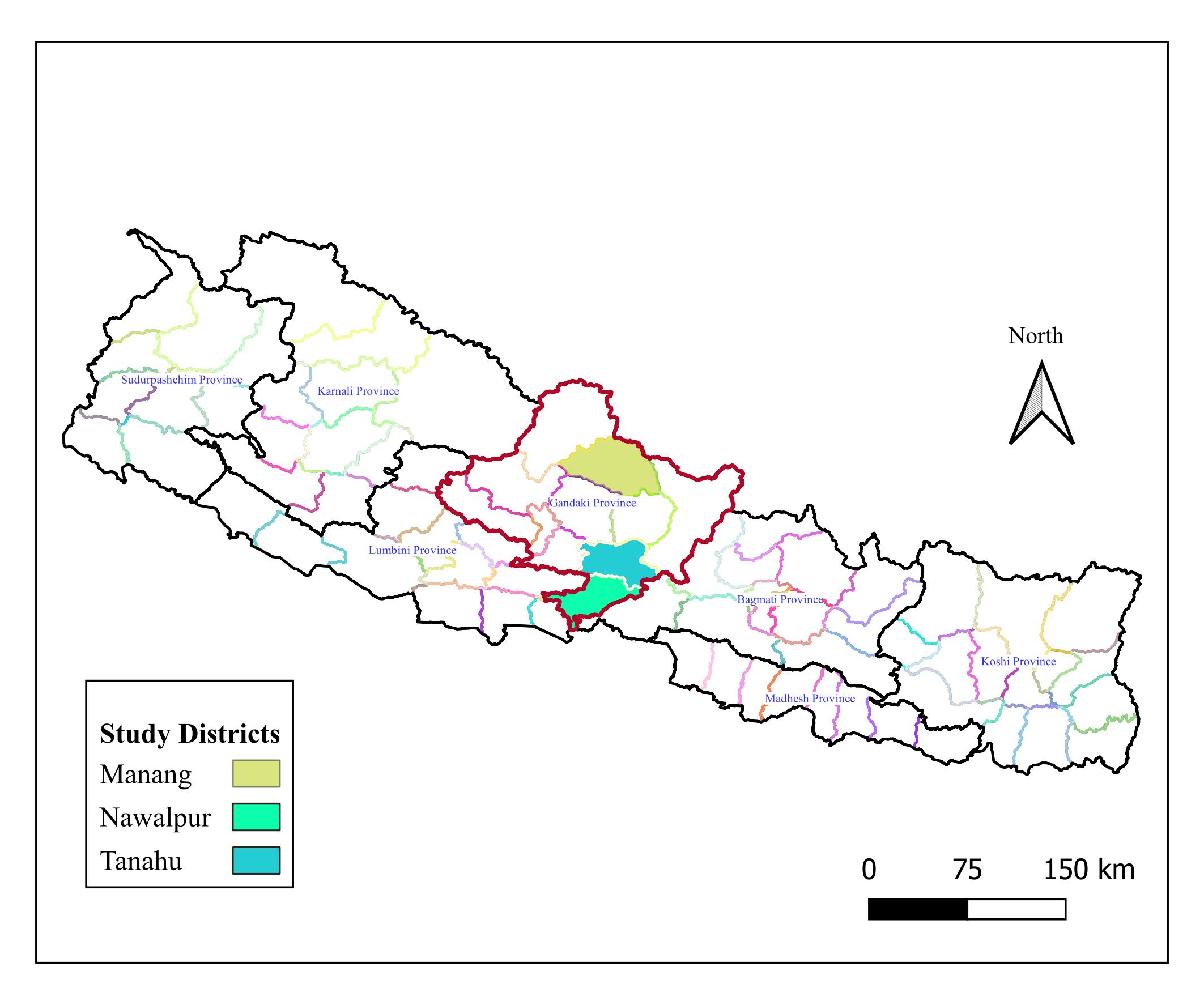

Supplement: S1 Fig — (TIF) [file pone.0323713.s002.tif]
