# Supplementary material for: Prevalence of family-based elder abuse and its associated factors in Gandaki Province of Western Nepal: A cross-sectional study
Source: PLoS One. 2025 May 14;20(5):e0323713. doi: 10.1371/journal.pone.0323713 (PMC12077681; doi:10.1371/journal.pone.0323713)
Supplement: Attachment — Submitted filename: Response to Reviewers.docx [file pone.0323713.s005.docx]

**Response to Reviewers**

Comments from Editorial Office

| **Feedback** | **Author response** |
| --- | --- |
| If there are ethical or legal restrictions on sharing a de-identified data set, please explain them in detail (e.g., data contain potentially identifying or sensitive patient information, data are owned by a third-party organization, etc.) and who has imposed them (e.g., a Research Ethics Committee or Institutional Review Board, etc.). Please also provide contact information for a data access committee, ethics committee, or other institutional body to which data requests may be sent. | Either of the following two can be communicated for data access. However, we already have deposited the data relevant to this study in the repository.  Bharat Kafle <bharatkafle29@gmail.com> |
| If there are no restrictions, please upload the minimal anonymized data set necessary to replicate your study findings to a stable, public repository and provide us with the relevant URLs, DOIs, or accession numbers. For a list of recommended repositories, please see  https://journals.plos.org/plosone/s/recommended-repositories. You also have the option of uploading the data as Supporting Information files, but we would recommend depositing data directly to a data repository if possible. | Below is the citation of the data repository where the relevant dataset is deposited.   Kafle, Bharat, and Shrestha, Aman. Elder Abuse in Gandaki Province of Western Nepal. Ann Arbor, MI: Inter-university Consortium for Political and Social Research [distributor], 2024-10-01. <https://doi.org/10.3886/E209442V1> |
| We note that your Data Availability Statement is currently as follows: [All relevant data are within the manuscript and its Supporting Information files.]   Please confirm at this time whether or not your submission contains all raw data required to replicate the results of your study. Authors must share the “minimal data set” for their submission. PLOS defines the minimal data set to consist of the data required to replicate all study findings reported in the article, as well as related metadata and methods (<https://journals.plos.org/plosone/s/data-availability#loc-minimal-data-set-definition>).  If your submission does not contain these data, please either upload them as Supporting Information files or deposit them to a stable, public repository and provide us with the relevant URLs, DOIs, or accession numbers. For a list of recommended repositories, please see https://journals.plos.org/plosone/s/recommended-repositories. | We have uploaded the dataset to OpenICPSR data repository. |

**Reviewer-1**

| **Feedback** | **Author response** |
| --- | --- |
| The authors have well-articulated the manuscript and addressed an important topic. This study brings attention to elder mistreatment, which is often concealed in the study area due to the prevailing culture of filial piety. | We appreciate your recognition that elder abuse may manifest differently in societies where filial piety is deeply ingrained. Your understanding captures the essence of our research, particularly given the significant gap in gerontology-focused studies on this topic in South Asia. We are grateful for your thoughtful insights. |
| **Section: Introduction** | |
| This section is well-written with adequate literature review. | Thank you for your positive feedback. |
| **Section: Methods** | |
| The authors have well-described methods using appropriate techniques and procedures. I have some minor comments, which are as follows: | We sincerely appreciate your positive and constructive feedback and have carefully addressed all the comments as outlined below. We believe these revisions have significantly enhanced the rigor and quality of our work. Please find our detailed responses to each point below. |
| Since this study was conducted during peak time of COVID-19 pandemic situation, I suggest the authors to briefly explain how the team handled this situation and managed to face-to-face interview 612 interviews. | Thank you for raising this important concern. We acknowledge that conducting face-to-face interviews during the COVID-19 pandemic poses significant logistical challenges and raises understandable concerns about participant safety. However, our data collection took place during a unique time frame: January 9, 2020 – February 26, 2020, when the pandemic was rapidly spreading worldwide, but Nepal recorded only its first case near the end of our data collection. As a result, the pandemic had not yet affected the country during our data collection period, and consequently, no restrictions or special COVID-19 precautions were in place for our fieldwork.  Since we have clearly outlined the data collection timeline in the study methodology, and no pandemic-related measures were required at that time, we are unsure of any additional details that need to be included in the manuscript. However, if you believe further clarification is necessary, please let us know what specific information would be helpful, and we would be happy to revise the manuscript accordingly. Thank you again for your valuable feedback. |
| Can you please mention one or two examples in the set of 21 questions of abuse? How were those questioned asked to the participants? | Thank you for your valuable feedback. We have added example questions to the manuscript and explicitly clarified that all responses were binary, either "Yes" or "No." Additionally, we have cited the previous literature that developed the tool, allowing readers access to detailed information. The following statements have been added to the section on outcome variables under the 'Study Variables' subsection of the Methods. We appreciate your suggestions to improve clarity for our readers.  *Older adults participating in the study were asked to report any instances of abuse by family members in the past three months using a set of 21 questions, each measured with a binary "Yes" or "No" response. ……………………………………………………………. Comprehensive details of the tool and its 12 core items can be found in a previous study [ref]. Some example questions include: "Have you been beaten by any members of your family?", "Does your family insult you in front of others?", and "Do you feel that your family members are uncommunicative or unresponsive towards you?"……………….*  Regarding your comment on data collection, we have explicitly mentioned in the Data Collection section that face-to-face oral interviews were conducted. To further enhance transparency, we have now added a statement under the Ethical Considerations section, clarifying that no proxies were permitted during the interviews. We hope these revisions address your concerns, and we appreciate your thoughtful feedback. |
| In the Data analyses section, the authors mentioned SAS version 9.04 while it should have been 9.4. I suggest the authors verify the version of SAS. | Thank you for pointing out this typo. We appreciate your attention to detail and have corrected it to ‘SAS 9.4’ in the revised manuscript. |
| The method section could end up with mentioning how you interpret the results, particularly for regression model, e.g. OR, and at level of ?? significance or mention the CI%. | Thank you for your suggestion. We made some adjustments to the paragraph to improve the coherence of the information and added additional details. The revised section at the end of the data analyses sub-section reads as follows:  *Odds ratios (OR) and 95% confidence intervals (95% CI) from both unadjusted and adjusted binary logistic regression models are presented in Table 3. These values indicate the odds of experiencing any form of elder abuse at different levels of the explanatory variables, both with and without controlling for covariates. Statistical significance was defined as a p-value less than 0.05.* |
| **Section: Results** | |
| Well-done with your results! I have minor comment, to start your results with participants’ profile first and then interpret the prevalence of elder abuse, followed by regression analyses. | Thank you for your suggestion. We have restructured the results section to improve clarity and flow. It now begins with the characteristics of the participants, followed by the prevalence of elder abuse and its subtypes. Next, we present the bivariate associations, and finally, we present the findings from the regression analyses. We appreciate your feedback. |
| **Section: Discussion** | |
| A brief summary of your results including the associated factors would be informative to the readers. | Thank you for bringing this to our attention. We agree that this is an important context that helps frame the discussion, and its omission was unintentional. In response, we have added a brief summary of the associated factors and the first paragraph of the discussion section now reads as:  " *This study examined elder abuse prevalence in Gandaki Province and identified associated factors. Over half of the participants reported experiencing abuse, with a significantly higher prevalence among females. Furthermore, participants who were 70 years old, reluctant to disclose health issues to family members, believed in traditional healers, or lived in nuclear households had higher odds of reporting elder abuse compared to their respective counterparts. Conversely, those living in rural areas, with formal education, or reporting good self-rated health had lower odds of experiencing elder abuse."* |
| Approximately 56% of the participants reported experiencing abuse. It is possible that this value is overestimated due to the pandemic situation, particularly regarding caregiver neglect (50.8%). The COVID-19 pandemic likely exacerbated these factors due to fears of infection, social distancing measures, job losses, and reduced services for older adults. I suggest authors to discuss these viewpoints as well. | Thank you for your thoughtful feedback. We would like to kindly reiterate that there was no impact of COVID-19 during the data collection process. Therefore, we do not believe the pandemic influenced the prevalence estimates in this study. However, we do agree that if data collection had occurred during the pandemic, there may have been potential for overestimation, but this scenario does not apply to our study.  In the introduction, we have also highlighted that several small-scale studies over the past years have consistently reported high prevalence of elder abuse in Nepal, ranging from approximately 47% to 66%. Caregiver neglect has been the most commonly identified form, with a prevalence of 57.5%. As this area of research is still emerging, we expect that more studies will be conducted across various settings in the country, leading to more precise prevalence estimates over time. In fact, our team is currently working on a systematic review and meta-analysis on this topic to consolidate findings from multiple studies.  We hope this clarification addresses your concerns. Since COVID-19 did not affect our study findings, we did not add specific details related to the pandemic in the revised manuscript. However, if you believe further clarification is needed, please let us know what specific information would be helpful, and we would be happy to revise the manuscript accordingly. Thank you again for your valuable input. |

**Reviewer-2**

| **Feedback** | **Author response** |
| --- | --- |
| Structured abstract is needed. | Thank you for this suggestion. We have structured our abstract accordingly. |
| In the introduction section, review of context specific literature- total elder population, policy and program context in Nepal, issues and challenges of elder population, rationale and significance of study is not coming through. Needs more lit review and synthesis with relevant research gaps for this research. | Thank you for your suggestion. First regarding the statistics on the total elder population , we would like to draw your attention to Supplementary Table 1, which provides detailed demographic and socioeconomic characteristics of the study districts, including the total elder population.  In response to your valuable feedback on issues and challenges of elder population, we have added the following paragraph in the introduction section on page 4 briefing the impact of elder abuse on older adults:  *Elder abuse inflicts serious and multifaceted consequences, resulting in a wide array of health-related issues that compromise the well-being of older adults and significantly diminish their overall quality of life (3). Furthermore, the effect of elder abuse is not bound only to individuals but also places a considerable financial, caregiving, and other burden on society and the country (4)*.  In response to your recommendations regarding the policy and program context in Nepal, we have added a paragraph to the introduction on page 5, which reads as follows:  *Nepal does not have a policy that specifically addresses issues on elder abuse. However, a few legislations, regulations, and guidelines are geared toward protecting older adults’ rights and preventing abuse against them. The Senior Citizen Act (2006) ensures social security, welfare, and safeguarding rights for older adults (13). Under this statute, anybody who abuses older adults might face criminal penalties. In addition, the country’s constitution and other laws also offer protection and recourse for older people who may be victims of abuse. For example, article 41 of the Constitution of Nepal (2015) has ensured the right of older adults to live with dignity and provisions of safety and social security (14); the Domestic Violence Act (2009) criminalizes all forms of abuse within a household (15). Aside from these efforts, Nepal government has made a few other sporadic efforts, such as introducing National Plan of Action for Senior Citizens in 2005 (16), enacting Social Security Act 2018 (17), and incorporating older adults’ issues in the sixteenth five-year national plan (18), none of which have, in and of themselves, been adequate to address issues of elder abuse in Nepal.*  We hope these additions provide the necessary context and enhances the manuscript. Thank you again for your valuable feedback. |
| It is unclear how sample was estimated and how they were selected in the data collection process. A flow chart can be useful to show them. | Thank you for your valuable feedback. We agree that the sampling method, particularly the local context, may be challenging to understand, especially for readers unfamiliar with the region. We appreciate your suggestion to incorporate a flowchart to enhance clarity for our readers.  Following your recommendation, we have included a flow diagram (Fig 1 – *attached separately per journal guidelines*) that visually illustrates the sampling procedure. Additionally, we have provided further clarification in S2 Table in the Supporting document which outlines the sampling technique in detail. We hope these additions makes the sampling process easier to follow. Thank you again for your thoughtful suggestion. |
| It is unclear how tool was designed, tested and ensure validity of the tool in Nepali context. | Thank you for your feedback. We used a pre-existing 21-item elder abuse tool (8,11), which was created after a thorough literature review, pre-testing, and validation. This tool has been applied in multiple studies conducted in Nepal, specifically in rural municipalities in Morang (eastern Nepal) and urban municipalities in Butwal (western Nepal). Furthermore, our operational definitions of the various types of elder abuse are available in S3 Table.  While testing the validity of the tool was beyond the scope of the current study, we assessed the internal consistency of the scale among our study participants. With a McDonald’s Omega score of 0.91, we found the tool to be highly reliable. These details are already included in the manuscript, but if there are any specific points you feel should be added for further clarity, we would be happy to revise it accordingly.  We sincerely appreciate your feedback as we believe that one potential area for future research could be a formal validity testing of this scale among our sample, but we see that as a separate future manuscript. |
| Major issue lies in the analysis output, for example, the analysis of prevalence of categories of variables should be row percentage than column percent while running analysis of experience of abuse. Total prevalence of abuse in Hill region is estimated 138*100/240=57.5%. if yes is included there is no need to write no % and numbers. | Thank you for your feedback. We have updated the bivariate analysis by switching from column to row percentages to enhance clarity. While we agree that including percentages and numbers for the 'No' category may not be essential, we chose to retain them for consistency and clarity. Please note that, to maintain the order, Table 2 from the original manuscript has been split into two tables: Table 1 now presents demographic characteristics, and Table 3 covers the bivariate associations. These were previously combined as Table 2 in the original submission. We appreciate your valuable input. |
| After careful analysis, result section needs revision. | Thank you. We revised the corresponding result narrative to reflect the changes. Please refer to the "Bivariate Association Between Participants' Characteristics and Their Experience of Abuse" subsection of the Results section for the updated narrative. |
| Once we have clear findings, discussion section needs focused analysis and interpretation linking with policy, practices, systems and services in the federalized context of Nepal. | Thank you for your feedback. Since the major findings remain unchanged following the suggested data analysis, we did not revise the discussion section based on the updates to Table 3. However, if you feel further adjustments are needed, please let us know, and we will be happy to revisit the manuscript. |
