# Supplementary material for: Prevalence of family-based elder abuse and its associated factors in Gandaki Province of Western Nepal: A cross-sectional study
Source: PLoS One. 2025 May 14;20(5):e0323713. doi: 10.1371/journal.pone.0323713 (PMC12077681; doi:10.1371/journal.pone.0323713)
Supplement: Attachment — Submitted filename: Revised Manuscript with TC_ Acad_edit_ 8 Nov 2024.docx [file pone.0323713.s004.docx]

# Prevalence of family-based elder abuse and its associated factor in Gandaki Province of western Nepal: A cross-sectional study

Bharat Kafle^1(¶,*)^, Aman Shrestha^2(¶)^, Saruna Ghimire^3^, Preeti Bhattarai^1^, Pratik Bhattarai^4^, Amod Kumar Poudyal^5^.

^1^School of Public Health, Karnali Academy of Health Sciences, Jumla, Karnali Province, Nepal

^2^Division of Gerontology, Department of Epidemiology & Public Health, School of Medicine, University of Maryland Baltimore, Maryland, United States of America

^3^Department of Sociology and Gerontology and Scripps Gerontology Center, Miami University, Oxford, Ohio, United States of America

^4^Oxford University Clinical Research Unit, Lalitpur, Bagmati Province, Nepal

^5^Central Department of Public Health, Institute of Medicine, Tribhuvan University, Maharajgunj, Kathmandu, Bagmati Province, Nepal

^*^Corresponding author

E-mail: [bharatkafle29@gmail.com](mailto:bharatkafle29@gmail.com) (BK)

^¶^These authors contributed equally to this work.

# Abstract

## Background

Elder abuse is a public health and human rights issue rarely discussed, observed, and reported in Nepal. Traditional values promoting filial piety hinder the acknowledgment and reporting of such abuse, particularly within family settings. This study included all three ecological regions as well as both urban and rural areas of Nepal to determine the prevalence and associated factors of family-based elder abuse.

## Methods

Participants were selected using multistage stratified cluster sampling. Older adults, 60 years and older, were interviewed across three districts, one each from three ecological regions in Gandaki Province. The final analytic sample was 612. A 17-item elder abuse scale assessed the outcome.

## Results

More than half of the participants were from Nawalpur-Tarai (57.2%), urban residents (68.1%), female (57.2%), without education (79.8%), and lived in multigenerational households (73.0%). The overall prevalence of elder abuse reported in this study was 56.4%, with caregiver neglect (50.8%) as the most prevalent sub-type. Except for financial abuse, the overall prevalence and subtypes of elder abuse were significantly higher in females than in males. In adjusted multivariable logistic regression, those who were female (odds ratio [OR]=2.56, 95% confidence interval [95%CI]: 1.64–4.01), older than 70 years (OR=1.53, 95%CI: 1.03–2.29), reluctant to disclose health issues to family members (OR=2.13, 95%CI: 1.36–3.34), believed in traditional healers (OR=1.89, 95%CI: 1.28–2.77) and lived in nuclear households (OR=1.85, 95%CI: 1.19–2.89) had increased odds of elder abuse. Those living in rural areas (OR=0.39, 95%CI: 0.25–0.61), having a formal education (OR=0.31, 95%CI: 0.10–0.95), and having good self-reported health (OR=0.23, 95%CI: 0.12–0.46) were associated with lower odds of elder abuse.

## Conclusions

Elder abuse is a critical issue that requires the immediate attention of policymakers and stakeholders, particularly with the growing older population and the changing traditional care system in Nepal.

**keywords:** senior, mistreatment, maltreatment, south Asia, family violence.

# Introduction

Elder abuse is a pervasive, deeply ingrained social issue impacting older people globally. The terminology and definition of abuse against older persons lack consistency in the literature. While several previous studies have used the terms “mistreatment” and “abuse” interchangeably, this study adopts “abuse” for consistency. Elder abuse, as a term, lacks a universally accepted definition but generally refers to any intentional or negligent act puts an older adult at risk, perpetrated by individuals who have control over, are responsible for, or have a trusted, caring relationship with the older adult (1). The World Health Organization has defined elder abuse as “a single or repeated act or lack of appropriate action, occurring within any relationship where there is an expectation of trust which causes harm or distress to an older person” (2). Such abuse, occurring in both institutional and community settings, can manifest in various forms, including physical, mental, sexual, or emotional, and extend to financial exploitation, acts of neglect, and abandonment, such as denying involvement in decision-making processes and restricting personal spaces (1). Elder abuse inflicts serious and multifaceted consequences, resulting in a wide array of health-related issues that compromise the well-being of older adults and significantly diminish their overall quality of life (3). Furthermore, the effect of elder abuse is not bound only to individuals but also places a considerable financial, caregiving, and other burden on society and the country (4). Globally, a systematic review and meta-analysis, including studies from 28 countries, revealed that one in six older adults reported experiencing abuse in community settings (5). In institutionalized settings, 64.2% of staff have admitted to abusing older adults (6). However, these studies also indicate that the actual prevalence of elder abuse may be much higher due to factors like underreporting and the lack of a standardized definition and measurement methods for elder abuse. Abuse towards older adults can result in severe physical and mental health issues, financial problems, and social consequences. Some of these include physical injuries, cognitive decline, depression, hospitalization, premature mortality, financial devastation, and forced placement in alternative care options (7).

Although recognized as a significant public health and human rights concern globally, abuse towards older people, specifically by family members, remains largely overlooked, especially in countries with traditional cultures and filial piety, such as Nepal, a South Asian country situated between India and China (8). On the one hand, elder abuse is perceived as a societal taboo in Nepal; on the other hand, the traditional belief systems that emphasize respect and care for older family members paradoxically create barriers to acknowledging, reporting, and addressing incidents of elder abuse (8,9). Consequently, there is a common belief that elder abuse is rare in Nepal. However, this conception is not supported by empirical evidence; in fact, several previous studies have revealed a higher prevalence of elder abuse in the country. Small-scale studies on elder abuse conducted in the last ten years have shown varying prevalence, ranging from 46.6% to 65.6% (8–11). With a maximum prevalence of 57.5%, caregiver neglect has been consistently found to be the most prevalent form of elder abuse in all of these four studies. Various forms of elder abuse reported in Nepal include accusations of witchcraft, caste-based discrimination, familial victimization, misuse of old-age allowances, negligence, sexual misconduct, and torture, often stemming from perceptions of older members as financial burdens on their families (12).

Nepal does not have a policy that specifically addresses issues of elder abuse. However, a few legislations, regulations, and guidelines are geared toward protecting older adults’ rights and preventing abuse against them. The Senior Citizen Act (2006) ensures social security, welfare, and safeguarding rights for older adults (13). Under this statute, anybody who abuses older adults might face criminal penalties. In addition, the country’s constitution and other laws also offer protection and recourse for older people who may be victims of abuse. For example, article 41 of the Constitution of Nepal (2015) ensures the right of older adults to live with dignity and provisions of safety and social security (14); the Domestic Violence Act (2009) criminalizes all forms of abuse within a household (15). Aside from these efforts, the government in Nepal has made a few other sporadic efforts, such as introducing the National Plan of Action for Senior Citizens in 2005 (16), enacting the Social Security Act 2018 (17), and incorporating older adults’ issues in the sixteenth five-year national plan (18), none of which have, in and of themselves, been adequate to address the problems of elder abuse in Nepal.

Although previous studies have shed light on the issue of elder abuse, they were limited by small sample size and geographical coverage. So far, no comprehensive national, provincial, or pooled prevalence study on elder abuse has been conducted in Nepal. The recent census data showing a significant increase in the older population of 60 years and above — from 6.4% in 2001 to 10.2% in 2021 — indicates Nepal's population is aging (19). This demographic shift implies that issues concerning older adults, including incidents of elder abuse, are likely to become more prominent, underscoring the need for greater attention to this often-underreported issue. To the authors’ knowledge, this study is the first to comprehensively examine elder abuse using representative samples from all three ecological regions of the Gandaki Province in Nepal. With the overarching aim of understanding elder abuse in Nepal, this study has two specific objectives: 1) to calculate the prevalence of elder abuse by family members and 2) to identify the factors associated with such abuse.

# Materials and methods

This study follows the STROBE (STrengthening the Reporting of OBservational Studies in Epidemiology) guidelines for its reporting purposes (20).

## Ethical consideration

This study obtained ethical approval from the Institutional Review Committee at the Institute of Medicine, Tribhuvan University (reference no. 194/(6-11) E2 076/077) and received written permission from the Social Development Ministry of Gandaki Province (reference no. 1019/075-076) in Nepal. Participants were informed about the study and its purpose, voluntary participation, and their rights to withdraw anytime. They provided written consent before their interviews, either through signatures or thumbprints. For participants unable to read, they discussed the consent forms with their trusted individuals before providing their consent. Interviews were conducted privately in the participants' homes, with arrangements made to ensure confidentiality and minimize interruptions. No proxy interviews were conducted.

## Study design and settings

Nepal recently adopted a seven-province administrative system, and this community-based cross-sectional study was conducted in Gandaki Province, also known as Province 4, located in western Nepal (S1 Fig). Gandaki Province was selected for this study due to its high proportion of older adults, the highest among all seven provinces (21,22), its ethnic diversity, which includes significant populations of both “Brahaman/Chhetri” and “Adivasi/Janajati,” and for having the second-highest distribution of ethnic minorities from “Dalit/Muslim” groups compared to other provinces (23). Additionally, Gandaki Province covers all three ecological regions of Nepal — mountains, hills, plains, or tarai — offering a substantial geographical and lifestyle diversity that mirrors the wide spectrum of ecological regions of Nepal. S1 Table presents various demographic and socioeconomic indicators for Gandaki Province and its study districts, comparing them to the national average using data from the 2021 census. Gandaki Province has a total population of 2,466,427, accounting for 8.5% of the national population (19). Approximately 13.5% of the population in this province are 60 years or older. The old-age dependency ratio in the province is 21.25, which is higher than the national old-age dependency ratio of 16.48 (19). Regarding development, the Human Development Index in Gandaki Province ranges from 0.48 to 0.58, closely matching the national average of 0.49 (23).

## Sample size and sampling

The sample size of 616 was calculated based on a 49.1% prevalence of elder abuse reported in a previous study (8), considering a 95% confidence interval, a cluster design effect of 1.5, a 5% non-response rate, and a stratification effect of two. The details of the sampling technique are illustrated in the S2 Table. Multistage stratified cluster sampling was used to select participants. In the first sampling stage, one district was randomly selected from each ecological region: Manang from the mountains, Tanahu from the hills, and Nawalpur, also called East Nawalparasi, from the tarai. Subsequently, using simple random sampling, one urban and one rural municipality were selected from the Nawalpur and Tanahu districts. However, in Manang, only one rural municipality was chosen randomly, as this district has no urban municipality. In the subsequent stage, within each selected municipality, wards (the smallest administrative units in Nepal) were randomly chosen as clusters or primary sampling units based on population proportionate to size. Then, within each ward, systematic random sampling was used to select every third household until the target sample size of 22 households per cluster was reached. The sampling flow diagram of the study is presented below (Fig 1).

**Fig 1. Sampling Procedure of the Study**.

*Note.* RM=rural municipality; UM=urban municipality; SRS=simple random sampling; PPS=probability proportional to size; KISH=a random sampling technique.

Only one older adult from each selected household was interviewed. For more than one eligible older adult living in the same household, only one was selected at random. The survey included older adults who were permanent residents in the study area, 60 years or above, and proficient in Nepali. Older adults with hearing or speech impairments that prevented effective communication were excluded from the study.

## Data collection

Using a semi-structured, paper-based questionnaire, 612 face-to-face oral interviews were conducted between January 9, 2020, and February 26, 2020. Initially in English, this questionnaire was translated into Nepali to facilitate data collection. Three co-authors in this manuscript and two local research assistants from Gandaki Province, all with prior experience in research data collection, were recruited for the fieldwork. They underwent a one-day training session that covered various aspects of the study, including its purpose, research protocols and procedures, ethical considerations, participant recruitment, interviewing techniques, data collection processes, and safety measures. A pilot testing of the survey instrument was carried out with 30 older adults in ward no. 26 of Kathmandu Metropolitan City. While the responses from this pilot test were excluded from the primary data analysis, they proved valuable for identifying and rectifying several sensitive language issues in the questionnaire. Subsequently, the data collected from the paper questionnaires were digitized by creating data entry panels in EpiData 3.1 (24).

## Study variables

### Outcome

Older adults participating in the study were asked to report any instances of abuse by family members in the past three months using a set of 21 questions, each measured with a binary "Yes" or "No" response. The abuse scale, developed for older adults in eastern Nepal, has been previously used to assess abuse among older adults in community settings within Nepal (8,11). Operational definitions of abuse and its various types are detailed in the S3 Table. The questionnaire covered various types of abuse, starting with an assessment of general types of elder abuse, followed by three items on physical abuse, five items each on psychological abuse and caregiver neglect, three items on financial abuse, and one item on sexual abuse. Comprehensive details of the tool and its 21core items can be found in a previous studies (8,11) . Some example questions include: "Have you been beaten by any members of your family?", "Does your family insult you in front of others?", and "Do you feel that your family members are uncommunicative or unresponsive towards you?" As this study focused on elder abuse within family settings, the three questions related to legal abuse were excluded, as they were contextually outside family settings. Furthermore, the initial question, “Have you experienced some form of mistreatment in the last three months?” was omitted, considering that similar information could be inferred from other items in the scale. In this study, elder abuse was defined as the occurrence of at least one of the five types of abuse captured in the tool: physical, psychological, financial, sexual abuse, and caregiver neglect. The internal consistency of the scale, which included the seventeen items used in this study, resulted in a high McDonald’s Omega score of 0.91, signifying the high reliability of this tool (25).

### Predictors

#### Sociodemographic factors

Sociodemographic factors included a range of variables, including study districts, municipality type, age, gender, ethnicity, marital status, and disability status. As described in the methods section above, the three randomly selected districts were Manang, Tanahu, and Nawalpur, representing the mountain, hill, and tarai regions in order. The classification of selected municipalities as “rural” or “urban” was based on the Nepal Gazette 2073, an official publication of the Government of Nepal (26). Age was a numeric variable that displayed a right-skewed distribution and thus was divided into “60 to 70 years” and “above 70 years.” Gender had two categories: “female” and “male.” Following the approach of previous studies (8,11), ethnicity was reclassified into three categories: “Brahaman/Chhetri,” “Adivasi/Janajati,” and “Dalit/Muslim” (27). The “Brahaman/Chhetri” included “Hill Brahaman/Chhetri” and “Tarai/Madhesi Brahaman/Chhetri.” The “Adivasi/Janajati” groups included “Newar,” “Hill Janajati,” and “Tarai Janajati.” Similarly, “Dalit/Muslim” included “Hill Dalit,” “Tarai/Madhesi Dalit,” and “Musalman.”

Marital status was recategorized into two groups: “married” and “without a partner”; the latter category included individuals who were “unmarried,” “widowed,” “divorced,” or “separated.” Self-reported disability status was defined following the definition provided in the schedule related to sub-section (1) of section 3 in Nepal Gazette 2074 (28). The definition follows: “A person with a disability means a person who has long-term physical, mental, intellectual or sensory disability or functional impairments or existing barriers that may hinder his or her full and effective participation in social life on an equal basis with others.” Participants were asked if they had “no disability,” “physical disability,” “visual impairments,” “hearing impairments,” “hearing and visual impairments,” “unable to speak,” “psychosocial disabilities,” “intellectual disability,” “hemophilia,” “autism,” or “multi-disability.” Participants with at least one disability were grouped as "disabled." Those without disabilities were grouped as "not disabled."

#### Socioeconomic factors

Socioeconomic factors included participants' education status and two family-related factors: (1) type of family ("nuclear" vs. "multigenerational"); and (2) monthly family income (converted to US$; the average exchange rate in 2020 was $1=NRs. 118.5106). Education was grouped into three categories: "no education," "non-formal education," and "formal education." "No education" referred to participants who could not read and write; those who went to classes or training outside of formal school settings and thus could read and write in Nepali were categorized as "non-formal education," while those who went to structured and systematic learning in schools were categorized as "formal education."

#### Social participation and interaction

Participation in social activities was assessed by asking participants if they got involved in any community-organized social activities and events within one year. Similarly, participation in community programs was assessed by asking whether they participated or gathered in any program organized by the community within the last week. Visits from relatives in the past month were measured by asking whether any relative had visited the participant within the last month. Participants could respond "yes" or "no" to each question.

#### Health-related factors

Healthcare utilization was assessed by asking participants if they had visited a medical institution in the past year, with binary responses of "yes" or "no." Behavioral health factors included two items: 1) whether participants believed in traditional healers and 2) their reluctance to disclose health issues to family members, both with binary responses of “yes” or “no.” Self-reported health status was measured by asking participants, "How do you rate your health condition today?" The possible responses were: "good,” "satisfactory,” "neutral,” "not satisfactory," and "poor." Due to low-frequency counts, related categories were merged to create a three-level self-rated health variable: "poor" (combining "poor" and "not satisfactory"), "good" (combining "good" and "satisfactory"), and "neutral." Healthcare access was assessed by asking if participants had access to the health facility to receive healthcare services. The responses were “yes” or “no.”

### Data analyses

Data from EpiData was imported into SAS 9.4 for analysis (29). In descriptive analyses (Table 1), categorical variables were presented as frequencies and percentages. The only continuous variable, monthly family income, was reported as the median and interquartile range due to its high right skew. Table 2 shows the prevalence of different types of elder abuse and their gender-specific prevalence and differences assessed using the Pearson Chi-square test of independence. Similarly, the bivariate relationships between abuse experience and categorical predictors were assessed using the Pearson Chi-square test of independence, while the Mann-Whitney test evaluated the relationship between elder abuse and the continuous predictor, monthly family income (Table 3).

All the variables described in Table 3 were assessed for multicollinearity using the variance influence factor (VIF) with a cutoff point 2.5 in the initial model (30). Notably, "Participation in social activities" (VIF=2.82) and "participation in community programs" (VIF=2.96) showed evidence of multicollinearity, with a high correlation (r=0.79). As a result, the variable with the higher VIF value (i.e., participation in community programs) was removed from the model to address multicollinearity concerns.

The best-fitting model, determined by minimizing the AIC criteria, excluded six variables: districts, ethnicity, marital status, monthly family income, participation in social activities, and healthcare access. Considering the distinctions between the three studied districts and the importance of ethnicity and marital status in defining and describing elder abuse in Nepali society (8,31,32), district, ethnicity, and marital status were deemed crucial and were retained in the final model. Therefore, the final adjusted model (Table 4) included district, municipality type, age, gender, ethnicity, marital status, disability status, education, family type, relative visiting within the last month, healthcare visit within the last year, belief in traditional healers, reluctance to disclose health issues to family members and self-rated health.

Concordance statistics (c-statistic), which range from 0.5 to 1 (with higher values indicating a better fit), were used to evaluate the model's goodness of fit. The c-statistic for the final adjusted model was 0.78, signifying that the final model demonstrated good predictive power (33). Furthermore, advanced diagnostics did not detect any influential observations (34). Both unadjusted and adjusted multivariable logistic regression models were conducted to identify the factors associated with elder abuse. Odds ratios (OR) and 95% confidence intervals from both unadjusted and adjusted multivariable logistic regression models are presented in Table 4. These values indicate the odds of experiencing any form of elder abuse at different levels of the predictors, both with and without controlling for covariates. Statistical significance was defined as a p-value less than 0.05.

# Results

## Characteristics of study participants

Table 1 below shows the overall characteristics of the study participants. More than half of the older adults were from the tarai region (57.2%), residents of urban municipalities (68.1%), aged between 60 and 70 years of age (56.7%), and were female (57.2%). Participants from “Brahaman/Chhetri” groups (41.2%) and “Adivasi/Janajati” (46.4%) comprised the majority, with only 12.4% belonging to “Dalit/Muslim.” About 63% were married, 44% reported disabilities, 80% did not have any education, and 73% lived in multigenerational households. The median monthly family income was $126.6 (IQR=185.6). More than half did not engage in social activities (58.2%) and community programs (59.5%), and about 21% had no recent visits from relatives. Approximately three-fourths (73.2%) sought healthcare, although more than half (50.8%) also believed in traditional healers. Notably, 27.9% were reluctant to disclose health issues to family, but only 15.3% rated their health as poor; 54.6% reported neutral health.

Table 1. Overall characteristics of study participants (N=612).

| **Variables** | **Frequency** | **Percentage** |
| --- | --- | --- |
| **Sociodemographic factors** | | |
| Study district | | |
| Tarai (Nawalpur) | 350 | 57.2 |
| Hill (Tanahu) | 240 | 39.2 |
| Mountain (Manang) | 22 | 3.6 |
| Municipality type | | |
| Urban | 417 | 68.1 |
| Rural | 195 | 31.9 |
| Age | | |
| 60-70 years | 347 | 56.7 |
| Above 70 years | 265 | 43.3 |
| Gender | | |
| Male | 262 | 42.8 |
| Female | 350 | 57.2 |
| Ethnicity | | |
| Brahaman/Chhetri | 252 | 41.2 |
| Adivasi/Janajati | 284 | 46.4 |
| Dalit/Muslim | 76 | 12.4 |
| Marital status | | |
| Married | 385 | 62.9 |
| ^1^Without a partner | 227 | 37.1 |
| Disability status | | |
| Without disability | 344 | 56.2 |
| With disability | 268 | 43.8 |
| **Socioeconomic factors** | | |
| Education | | |
| No education | 488 | 79.8 |
| Non-formal education | 98 | 16.0 |
| Formal education | 26 | 4.2 |
| Type of family | | |
| Multigenerational | 447 | 73.0 |
| Nuclear | 165 | 27.0 |
| ^2^Monthly family income in US$ (Median±IQR) | 126.6 | 185.6 |
| **Social participation and interaction** | | |
| ^2^Participation in social activities | | |
| No | 356 | 58.2 |
| Yes | 256 | 41.8 |
| ^3^Participation in community programs | | |
| No | 364 | 59.5 |
| Yes | 248 | 40.5 |
| Visits from relatives in the past month | | |
| Yes | 484 | 79.1 |
| No | 128 | 20.9 |
| **Health related factors** | | |
| Healthcare visit within the last year | | |
| Yes | 448 | 73.2 |
| No | 164 | 26.8 |
| Believe traditional healer | | |
| Yes | 311 | 50.8 |
| No | 301 | 49.2 |
| Reluctance to disclose health issues to family members | | |
| No | 441 | 72.1 |
| Yes | 171 | 27.9 |
| Self-Rated health | | |
| Poor | 94 | 15.3 |
| Neutral | 334 | 54.6 |
| Good | 184 | 30.1 |
| ^2^Healthcare access | | |
| Yes | 489 | 79.9 |
| No | 123 | 20.1 |

*Note:* Md=median, IQR=inter-quartile range; ^1^includes unmarried, widowed, divorced and separated; ^2^ excluded in regression based on AIC criteria; ^3^ excluded in regression due to multicollinearity.

## Prevalence of elder abuse: Overall and sub-types

Table 2 below presents data on the prevalence of family-based elder abuse in Gandaki Province, Nepal. The overall prevalence of elder abuse was 56.4% (95%CI: 52.34-60.34). Caregiver neglect was the most reported form of elder abuse (50.8%), followed by psychological abuse (35.5%), while sexual abuse (4.3%) was the least reported type. The prevalence of overall abuse and all its subtypes, except for financial abuse, was statistically significant and higher among females than males (Table 2). Table 2. Overall and gender-specific prevalence of various forms of elder abuse (N=612).

| **Type of abuse** | **Prevalence (%) (95%CI)** | | | **p-value** |
| --- | --- | --- | --- | --- |
|  | **Total** | **Male** | **Female** |  |
| Physical | 22.7  (19.45-26.24) | 10.7  (7.22-15.07) | 31.7  (26.87-36.87) | **<0.001^***^** |
| Psychological | 35.5  (31.66-39.39) | 21.4  (16.57-26.84) | 46.0  (40.69-51.38) | **<0.001^***^** |
| Caregiver neglect | 50.8  (46.78-54.85) | 35.9  (30.07-42.01) | 62.0  (56.91-67.09) | **<0.001^***^** |
| Financial | 11.9  (9.47-14.76) | 11.5  (7.86-15.94) | 12.3  (9.04-16.19) | 0.752 |
| Sexual | 4.3  (2.79-6.16) | 2.3  (0.84-4.92) | 5.7  (3.53-8.69) | **0.038^*^** |
| **Overall** | **56.4  (52.34-60.34)** | **42.8  (36.68-48.98)** | **66.6  (61.36-71.50)** | **<0.001^***^** |

*Note:* ^*^p<0.05, ^**^p<0.01, ^***^p<0.001, indicating statistical significance based on the Pearson Chi-square Test of Association between abuse and gender.

## Bivariate association between participants’ characteristics and their experience of abuse

Table 3 below depicts the characteristics of the study participants and bivariate comparisons based on abuse experience. Significant predictors under bivariate tests were municipality type, gender, marital status, disability status, education, monthly family income, participation in social activities and community programs, visits from relatives in the past month, healthcare visit within the last year, believe traditional healer, reluctance to disclose health issues to family members and self-rated health.

The prevalence of elder abuse was significantly higher among those who lived in urban municipalities (61.6%), were females (66.6%), without a partner (66.1%), and with disability (64.2%). With education, elder abuse was found to significantly drop from no education at 60.7% to formal education at 19.2%. Abuse was higher among participants who did not participate in social activities (63.5%) or community programs (62.9%) and those who did not have visits from the relatives in the past month (67.2%). Participants who were reluctant to disclose health issues to family members faced significantly higher abuse (76.0%).

Table 3. Bivariate analyses of characteristics of study participants (N=612).

| **Variables** | **Experienced abuse?** | | **p-value** |
| --- | --- | --- | --- |
|  | **Yes (n,%)** | **No (n,%)** |  |
|  | 34 (56.4%) | 267 (43.6%) |  |
| **Sociodemographic factors** | | | |
| Study District | | | 0.776 |
| Tarai (Nawalpur) | 196 56.0) | 154 (44.0) |  |
| Hill (Tanahu) | 138 (57.5) | 102 (42.5) |  |
| Mountain (Manang) | 11 (50.0) | 11 (50.0) |  |
| Municipality type | | | **<0.001^***^** |
| Urban | 257 (61.6) | 160 (38.4) |  |
| Rural | 88 (45.1) | 107 (54.9) |  |
| Age | | | 0.114 |
| 60-70 years | 186 (53.6) | 161 (46.4) |  |
| Above 70 years | 159 (60.0) | 106 (40.0) |  |
| Gender | | | **<0.001^***^** |
| Male | 112 (42.8) | 150 (57.2) |  |
| Female | 233 (66.6) | 117 (33.4) |  |
| Ethnicity | | | 0.674 |
| Brahaman/Chhetri | 143 (56.8) | 109 (43.2) |  |
| Adivasi/Janajati | 156 (54.9) | 128 (45.1) |  |
| Dalit/Muslim | 46 (60.5) | 30 (39.5) |  |
| Marital status | | | **<0.001^***^** |
| Married | 195 (50.6) | 190 (49.4) |  |
| ^1^Without a partner | 150 (66.1) | 77 (33.9) |  |
| Disability status | | | **0.001^**^** |
| Without disability | 173 (50.3) | 171 (49.7) |  |
| With disability | 172 (64.2) | 96 (35.8) |  |
| **Socioeconomic factors** | | | |
| Education | | | **<0.001^***^** |
| No education | 296 (60.7) | 192 (39.3) |  |
| Non-formal education | 44 (44.9) | 54 (55.1) |  |
| Formal education | 5 (19.2) | 21 (80.8) |  |
| Type of family | | | 0.142 |
| Multigenerational | 244 (54.6) | 203 (45.4) |  |
| Nuclear | 101 (61.2) | 64 (38.8) |  |
| ^2^Monthly family income in US$ (Median±IQR) | 109.7 (194.1) | 168.8 (210.9) | **<0.001^***u^** |
| **Social participation and interaction** | | | |
| ^2^Participation in social activities | | | **<0.001^***^** |
| No | 226 (63.5) | 130 (36.5) |  |
| Yes | 119 (46.5) | 137 (53.5) |  |
| ^3^Participation in community programs | | | **<0.001^***^** |
| No | 229 (62.9) | 135 (37.1) |  |
| Yes | 116 (46.8) | 132 (53.2) |  |
| Visits from relatives in the past month | | | **0.006^**^** |
| Yes | 259 (53.5) | 225 (46.5) |  |
| No | 86 (67.2) | 42 (32.8) |  |
| **Health related factors** | | | |
| Healthcare visit within the last year | | | **<0.001^***^** |
| Yes | 276 (61.6) | 172 (38.4) |  |
| No | 69 (42.1) | 95 (57.9) |  |
| Believe traditional healer | | | **<0.001^***^** |
| Yes | 196 (63.0) | 115 (37.0) |  |
| No | 149 (49.5) | 152 (50.5) |  |
| Reluctance to disclose health issues to family members | | | **<0.001^***^** |
| No | 215 (48.8) | 226 (51.2) |  |
| Yes | 130 (76.0) | 41 (24.0) |  |
| Self-Rated health | | | **<0.001^***^** |
| Poor | 77 (81.9) | 17 (18.1) |  |
| Neutral | 187 (56.0) | 147 (44.0) |  |
| Good | 81 (44.0) | 103 (56.0) |  |
| ^2^Healthcare access | | | 0.497 |
| Yes | 279 (57.1) | 210 (42.9) |  |
| No | 66 (53.7) | 57 (46.3) |  |

*Note:* ^*^p<0.05, ^**^p<0.01, ^***^p<0.001; ^1^includes unmarried, widowed, divorced and separated; ^2^excluded in regression based on AIC criteria; ^3^excluded in regression due to multicollinearity; IQR: inter-quartile range; ^u^P-value from Mann-Whitney test, all others are from Pearson Chi-square test.

## Factors associated with elder abuse: Regression analyses

The results of the multivariable logistic regression analysis for factors associated with family-based elder abuse are presented in Table 4. After adjusting for covariates, residing in a rural municipality was associated with 61% lower odds of elder abuse than living in an urban municipality [OR=0.39, 95%CI: 0.25–0.61]. Similarly, older females had 2.56 times the odds of experiencing abuse than older males [95%CI: 1.64–4.01]. Those with disabilities reported 63% higher odds of abuse than those without disabilities [OR=1.63, 95%CI: 1.10–2.42]. In the unadjusted model, education showed significance for both formal and non-formal categories. However, only the formal education category retained significance when controlled for covariates and was associated with 69% reduced odds of experiencing abuse [OR=0.31, 95%CI: 0.10–0.95]. Older adults living in nuclear families reported 85% higher odds of abuse compared to those living in multigenerational families [OR=1.85, CI: 1.19–2.89].

Table 4. Unadjusted and adjusted odds ratio (OR) for factors associated with elder abuse using multivariable logistic regression.

| **Characteristics** | **Unadjusted OR (95%CI)** | **^1^Adjusted OR (95%CI)** |
| --- | --- | --- |
| **Sociodemographic factors** | | |
| **Study district (ref= “Tarai (Nawalpur)”** | | |
| Hill (Tanahu) | 1.06 (0.76-1.48) | 0.93 (0.61-1.43) |
| Mountain (Manang) | 0.79 (0.33-1.86) | 1.54 (0.55-4.28) |
| **Municipality type (ref= "Urban")** | | |
| Rural | **0.51 (0.36-0.72)^***^** | **0.39 (0.25-0.61)^***^** |
| **Age** (ref= "60 to 70 years") | | |
| Above 70 years | 1.30 (0.94-1.80) | **1.53 (1.03-2.29)^*^** |
| **Gender** (ref= "Male") | | |
| Female | **2.67 (1.92-3.71)^***^** | **2.56 (1.64-4.01)^***^** |
| **Ethnicity** (ref= "Brahaman/Chhetri") | | |
| Adivasi/Janajati | 0.93 (0.66-1.31) | 1.02 (0.67-1.55) |
| Dalit/Muslim | 1.17 (0.69-1.97) | 0.96 (0.52-1.78) |
| **Marital status** (ref= "Married") | | |
| ^2^Without a partner | **1.90 (1.35-2.67)^***^** | 1.19 (0.78-1.82) |
| **Disability status** (ref= "Without disability") | | |
| With disability | **1.77 (1.28-2.46)^***^** | **1.63 (1.10-2.42)^*^** |
| **Socioeconomic factors** | | |
| **Education** (ref= "No education") | | |
| Non-formal education | **0.53 (0.34-0.82)^**^** | 1.09 (0.62-1.90) |
| Formal education | **0.15 (0.06-0.42)^***^** | **0.31 (0.10-0.95)^*^** |
| **Type of family** (ref= "Multigenerational") | | |
| Nuclear | 1.31 (0.91-1.89) | **1.85 (1.19-2.89)^**^** |
| **Social participation and interaction** | | |
| **Visits from relatives in the past month** (ref= "Yes") | | |
| No | **1.78 (1.18-2.68)^**^** | **1.64 (1.01-2.70)^*^** |
| **Health related factors** | | |
| **Healthcare visit within the last year** (ref= "Yes") | | |
| No | **0.45 (0.32-0.65)^***^** | 0.66 (0.43-1.01) ^ǂ^ |
| **Believe traditional healer** (ref= "No") | | |
| Yes | **1.74 (1.26-2.40)^***^** | **1.89 (1.28-2.77)^**^** |
| **Reluctance to disclose health issues to family members** (ref= "No") | | |
| Yes | **3.33 (2.24-4.96)^***^** | **2.13 (1.36-3.34)^**^** |
| **Self-Rated health** (ref= "Poor") | | |
| Neutral | **0.28 (0.16-0.50)^***^** | **0.31 (0.17-0.59)^***^** |
| Good | **0.17 (0.09-0.32)^***^** | **0.23 (0.12-0.46)^***^** |

*Note.* ^*^p<0.05, ^**^p<0.01, ^***^p<0.001, ^ǂ^approached significance; ^1^The model is mutually adjusted for all the variables presented in Table 3; ^2^includes unmarried, widowed, divorced and separated.

Among health-related factors, believing in a traditional healer, reluctance to disclose health issues to family members, and self-rated health showed highly significant associations with elder abuse after controlling for covariates. Older adults who believed in a traditional healer reported 1.89 times the odds of abuse compared to those who did not believe [CI=1.28–2.77]. Participants who were reluctant to disclose their health issues to family members had more than twice the increased odds of abuse compared to those who openly discussed their health issues [OR: 2.13, CI: 1.36-3.34]. Regarding self-rated health, older adults who rated their health as neutral had 69% lower odds of experiencing elder abuse [OR=0.31, CI: 0.17–0.59], while those who rated their health as good had 77% lower odds [OR=0.23, CI: 0.12–0.46], in comparison to older adults who rated their health as poor.

# Discussion

This study examined the prevalence of elder abuse in Gandaki Province and identified associated factors. Over half of the participants reported experiencing abuse, with a significantly higher prevalence among females. Furthermore, Participants who were over 70 years old, reluctant to disclose health issues to family members, believed in traditional healers, or lived in nuclear households had higher odds of reporting elder abuse compared to their respective counterparts. Conversely, those living in rural areas, with formal education, or reporting good self-rated health had lower odds of experiencing elder abuse.

The high prevalence of abuse reported by participants is consistent with previous cross-sectional studies in Nepal, which reported prevalences ranging from 46.6% to 65.6% (8–11,35). In contrast, a 2017 systematic review and meta-analysis on elder abuse in community settings estimated the global prevalence of elder abuse at 15.7% (5). This indicates that the burden of elder abuse found in this study is approximately 3.6 times higher than the global average. Additionally, recent literature from neighboring countries has reported varying elder abuse prevalence, ranging from 0.2% to 64% in China and 5% to 50.0% in India (36,37). Differences in prevalence among studies may arise from cross-national socio-cultural variations, but they also highlight potential discrepancies in methodology and the tools used to measure elder abuse. Notably, the assessment tools used in studies conducted in Nepal lacked validation within the local context.

Despite historically being a covert issue, this study’s findings, along with earlier research from Nepal, highlight the severity and pervasiveness of elder abuse in the country. The elevated prevalence of elder abuse in Nepal may also stem from cultural values such as filial piety and the typical multigenerational family structures, where older family members often rely on their adult children for care without receiving compensation (11). This can result in financial strain and heightened caregiving demands on families. Furthermore, the government of Nepal offers limited support for its older population, with existing welfare schemes providing minimal assistance. Also, institutional care for older adults is scarce in the country due to the cultural principle of filial piety, which mandates that adult children take care of their older parents legally and morally. The few existing facilities tend to be expensive, making them unaffordable for most older adults. Moreover, there is a strong stigma associated with seeking institutional care, as it is perceived as a sign of being abandoned by one's family, further discouraging older adults from accessing such services. Consequently, older family members primarily rely on their families for care without compensation. Daughters-in-law typically take on the primary role of providing daily tasks and personal assistance, while sons focus on ensuring financial stability (38). This traditional caregiving structure burdens family members significantly, potentially resulting in financial strain and intensified caregiving demands. In line with the theorized strain plausibility, neglect by family caregivers was the most common form of elder abuse in this study, accounting for 50.8% of cases, consistent with previous findings from Nepal (8–11). The increased dependency and declining financial autonomy in old age align with the political-economic theory of elder abuse (39), which states that older adults become vulnerable to potential abuse from family members as their role in the family shifts following retirement from the workforce.

Abuse was reported more frequently by older individuals who were above 70 years compared to those between 60 and 70 years, a trend supported by prior studies from Nepal (40) and a scoping review of international research indicating an increase in elder abuse with age (4). This finding may be explained by a decline in general health and well-being, as well as functional limitations that come with aging, leading to increased dependence on caregivers for daily activities and subsequently placing an increased burden on them, which may contribute to instances of abuse. Consistent with this explanation, this study also found that both disability and poor self-reported health—measures of physical functioning and overall health—were associated with higher odds of experiencing abuse. The findings align with a previous study from Nepal that found older adults with disabilities were approximately 12 times more vulnerable to abuse (40). Similarly, older individuals who rated their health higher had lower odds of elder abuse, echoing findings from an Indian longitudinal study that linked self-reported poor health with a 64% higher risk of elder abuse (41). Taken together, the burden of caregiving, particularly when required without sufficient support and financial compensation, as in Nepal, can lead to a rise in instances of abuse.

In this study, older females had 2.56 times the odds of experiencing elder abuse than older males (95%CI: 1.64–4.01); two earlier studies from Nepal support these findings (9,10). However, the literature on gender differences in elder abuse presents mixed results in both Nepal and global settings. Some research from Nepal reports higher abuse among older males (3,31), while two other studies found no statistically significant gender differences in abuse experiences (8,11). Similarly, a 2017 meta-analysis found a higher prevalence of elder abuse among females in Asia and the Pacific, although the association was not statistically significant (5). Although most studies suggest a trend of increased abuse among older women, the lack of statistical significance may be due to small sample sizes, as the meta-analysis included only 52 studies, and the two prior non-significant results from Nepal were derived from small-scale studies with only 212 and 339 participants. The observed gender disparity in abuse experiences may be attributed to patriarchal norms in Nepali families and is supported by feminist theories of elder abuse. The feminist theory of elder abuse explains this phenomenon through the perspective of traditional power dynamics within families and societies (39). Within this framework, it is observed that older women often wield less influence and authority, contributing to their increased vulnerability to abuse. The age gap between married couples is common in Nepal, where women tend to be younger than their male partners. This trend is shaped by long-standing cultural traditions, societal perceptions, and individual choices. In most cases, women assume caregiving roles for their husbands in their later years, but upon their husbands' passing, they find themselves reliant on their adult children for support. Consequently, Nepali women may encounter increased vulnerability, particularly considering their longer life expectancy than their male counterparts. Furthermore, it is unfortunate that Nepali society has historically undervalued women’s contributions, perpetuating their perception as a dependent population throughout their life journey. This high dependence may contribute to the elevated prevalence of abuse among older women in Nepal.

Two related findings—higher odds of abuse in urban areas and within nuclear families—may be correlated due to the tendency for households in urban areas to adopt more nuclear family structures. Although there is a lack of comparative studies on urban-rural differences in elder abuse in Nepal, the findings from this study represent a pioneering step in assessing these variations in reported abuse experiences. Research conducted in Canada and Nigeria corroborates the findings of this study (4). However, the findings from India, which shares a similar socio-cultural context with Nepal, consistently reported higher elder abuse in rural settings (41,42) and thus do not support the conclusion of this study. Nevertheless, a study from India reported physical abuse higher among rural residents, while neglect and psychological abuse were higher among urban residents (43). With urban migration emerging as a recent phenomenon in Nepal, older adults often relocate to where their children secure employment, exposing them to potential abuse in unfamiliar surroundings. Despite previous studies in India indicating lower instances of elder abuse in urban settings attributed to higher levels of education among older populations(44), only 4% of participants in this study had received formal education. Further, although there is a potential interaction between family type and urban-rural settings that could influence abuse experiences, the moderation analysis in this study did not yield significant results. Thus, this area warrants further exploration in future research, particularly in light of the recent reclassification of urban areas in Nepal. Recently, the urban area coverage defined in 2017 was 66.08%, which was only 17% in 2011, and this reclassification fails to adhere to internationally accepted standards of defining urban areas (45), suggesting a potential misclassification between urban and rural municipalities.

Abuse experiences reported in this study are more common in nuclear family settings, aligning with earlier findings in Nepal that smaller family sizes are associated with a higher report of abuse (40). A cross-country study from Bangladesh with similar family structures supports these conclusions (46), suggesting that strong family solidarity, filial piety, and collectivism may contribute to lower elder abuse in multigenerational households. Additionally, in multigenerational family settings, financial and caregiving responsibilities for older family members are shared among multiple family members, which helps to alleviate financial stress and caregiver burnout, subsequently reducing the risk of abuse. However, larger household sizes may also result in more family conflicts, potentially increasing the risk of elder abuse. Therefore, this complex area warrants further investigation in future research.

Although distinct, the findings on the significant association of education and belief in traditional healers could be explained with a common underlying role of awareness and decision-making. Regarding education, this study highlights the significant role of education in lowering the elder abuse experience. Those with formal education experienced 69% lower odds of facing elder abuse. Prior studies in Nepal also support the strong linkage between education and reduced elder abuse (10,11). For instance, a study by Yadav and colleagues found 69% higher odds of elder abuse among older individuals who were illiterate compared to those with education (11). Education thus can serve as a vital intervention, enabling individuals to recognize mistreatment and access legal assistance when necessary. Higher levels of education offer individuals better access to resources, information, support systems, and coping mechanisms, which can help reduce overall risk and vulnerability to abuse. Education also empowers individuals, contributing to a lower likelihood of experiencing abuse. This study uncovered an interesting link between elder abuse and belief in traditional healers, with older adults who trusted these healers experiencing higher rates of abuse. The relationship between elder abuse and faith in traditional healing practices has not been extensively studied, making direct comparisons with existing research challenging. However, this trend may be associated with the fact that older adults who have limited education often depend on traditional healers, reflecting the same underlying factors that link abuse to a lack of education (47).

Reluctance to share health concerns with family members, which was significantly linked to elder abuse, poses a notable health risk for older adults in Nepal and can lead to serious health problems. This hesitation may stem from older adults' desire to avoid burdening their children or reflect deeper trust issues and internal family conflicts. When older parents do not disclose their health problems to family members, it can result in misunderstandings, unrealistic expectations, and a lack of empathy, potentially creating conditions where neglect and abuse may thrive.

## Limitations and strengths

The cross-sectional nature of the data in this study limits its ability to make definitive conclusions about causality. Since elder abuse was evaluated based on self-reported instances from the past three months, there is a possibility of recall and information bias. Although the reliability score was high in this study, the tool used to measure elder abuse was not validated, which could affect the study's overall rigor. Additionally, the study did not measure the frequency or severity of the abuse experienced by older individuals within their families. Future research should prioritize using validated tools to assess elder abuse, as well as redefine elder abuse criteria to include measures of its frequency or severity.

This study represents a pioneering effort, covering all three ecological regions of Nepal and employing a rigorous sampling approach. To the author’s knowledge, this study is the first study in Nepal to examine the disparities in elder abuse between urban and rural environments.

## Conclusions

This study provides valuable insight into the higher prevalence of elder abuse in Gandaki province, with caregiver neglect appearing as the most common abuse type. Despite being a sensitive societal issue, the findings emphasize the importance of openly discussing and raising awareness about elder abuse in family settings. Abuse can severely impact the health and well-being of older adults, highlighting the need for caregiver education to increase awareness and understanding of the issue. Additionally, interventions should target modifiable risk factors such as caregiver burden, anxiety, and lack of social support while providing respite care and counseling services to prevent abuse and improve older adults' outcomes. Healthcare professionals should regularly assess for signs of abuse, caregiver burden, anxiety, and strained family relationships when interacting with older adults and their caregivers.

Public awareness campaigns are necessary to inform the public about the prevalence, risk factors, and consequences of elder abuse by family members. Promoting positive attitudes towards aging and challenging ageist stereotypes can also help prevent abuse. The study stresses the urgency of establishing and enforcing a comprehensive legal framework to combat issues of elder abuse effectively. While creating policies to protect older adults from abuse is essential, their successful implementation is even more crucial, though this is often lacking in countries like Nepal due to political instability and economic challenges. However, there is hope as the United Nations outlines five priorities to combat elder abuse within the decade of healthy aging from 2021 to 2030, and this study offers baseline information to guide federal and provincial governments and stakeholders in that effort.

# Conflict of Interest

The authors declare no conflict of interest.

# Funding

Not applicable.

# Author Contributions

AS, BK, PB^1^, and SG conceptualized the study; BK, PB^1^, and PB^2^ were responsible for field work; AS, BK, and SG analyzed the data and interpreted the findings; AS, BK, and SG wrote the first draft of the paper; all co-authors made critical revisions of the manuscript and approved the final version of the manuscript.

*Note.* PB^1^: Preeti Bhattrai; PB^2^: Pratik Bhattarai.

# Acknowledgments

We acknowledge the support from the Social Development Ministry and the local-level authorities in Gandaki province throughout this study. We are also deeply thankful to all the participants who generously contributed their time and insights to this research endeavor.

# References

1.	Cooper C, Selwood A, Livingston G. The prevalence of elder abuse and neglect: A systematic review. Age and Ageing. 2008;37(2):151–60. doi:10.1093/ageing/afm194

2.	World Health Organization. The Toronto declaration on the global prevention of elder abuse. Geneva: WHO [Internet]. 2002 [cited 2023 Apr 3];3. Available from: https://eapon.ca/wp-content/uploads/2021/09/toronto_declaration_en.pdf

3.	Joshi MR, Chalise HN. Elderly abuse and quality of life: A study of community living older people of Nepal. Journal of Medical Evidence. 2021;2(2):113-119. doi: 10.4103/JME.JME_140_20

4.	Pillemer K, Burnes D, Riffin C, Lachs MS. Elder abuse: Global situation, risk factors, and prevention strategies. Gerontologist. 2016;56:S194–205. doi: 10.1093/geront/gnw004

5.	Yon Y, Mikton CR, Gassoumis ZD, Wilber KH. Elder abuse prevalence in community settings: A systematic review and meta-analysis. The Lancet Global Health. 2017;5(2):e147–56. doi: 10.1016/S2214-109X(17)30006-2

6.	Yon Y, Ramiro-Gonzalez M, Mikton CR, Huber M, Sethi D. The prevalence of elder abuse in institutional settings: A systematic review and meta-analysis. The European Journal of Public Health. 2019 Feb 1;29(1):58. doi: https://doi.org/10.1093/eurpub/cky093

7.	Yunus RM, Hairi NN, Choo WY. Consequences of elder abuse and neglect: A systematic review of observational studies. Trauma Violence Abuse. 2019 ;20(2):197–213. doi: 10.1177/1524838017692798

8.	Yadav UN, Paudel G. Prevalence and associated factors of elder mistreatment: A cross sectional study from urban Nepal. Age and Ageing. 2016;45(5):609–14. doi: 10.1093/ageing/afw112

9.	Adhikari Baral M, Khatri Chhetri B, Bhandari P. Abuse of older adults before moving to old age homes in Pokhara Lekhnath Metropolitan City, Nepal: A cross-sectional study. PLoS One. 2021;16(5):e0250639. [doi](https://doi): 10.1371/journal.pone.0250639

10.	Acharya SR, Suman BK, Pahari S, Shin YC, Moon DH. Prevalence of abuse among the elderly population of Syangja, Nepal. BMC Public Health. 2021 ;21(1):1–9. doi: 10.1186/s12889-021-11417-0

11.	Yadav UN, Tamang MK, Paudel G, Kafle B, Mehta S, Sekaran VC, et al. The time has come to eliminate the gaps in the under-recognized burden of elder mistreatment: A community-based, cross-sectional study from rural eastern Nepal. PLoS One. 2018 Jun 1;13(6):e0198410. doi: 10.1371/journal.pone.0198410

12.	Upreti M. Elder abuse and mistreatment in Nepal. In: Shankardass MK, editor. International handbook of elder abuse and mistreatment [Internet]. 1st ed. Springer, Singapore; 2020 [cited 2023 Apr 3]. p. 385–403. Available from: https://link.springer.com/chapter/10.1007/978-981-13-8610-7_22

13.	Government of Nepal. Nepal Law Commission. 2006 [cited 2023 Apr 3]. Senior Citizens Act, 2063 (2006). Available from: https://www.lawcommission.gov.np/en/wp-content/uploads/2018/10/senior-citizens-act-2063-2006.pdf

14.	Government of Nepal. Nepal Law Commission. 2015 [cited 2023 Apr 3]. The Constitution of Nepal. Available from: https://lawcommission.gov.np/en/wp-content/uploads/2021/01/Constitution-of-Nepal.pdf

15.	Government of Nepal. Nepal Law Commission. 2009 [cited 2023 Apr 3]. Domestic violence (offence and punishment) act 2066 (2009). Available from: https://www.lawcommission.gov.np/en/wp-content/uploads/2018/10/domestic-violence-crime-and-punishment-act-2066-2009.pdf

16.	Government of Nepal. National Plan of Action for Senior Citizens 2005 [Internet]. 2005 [cited 2023 Apr 3]. Available from: https://nepal.unfpa.org/sites/default/files/pub-pdf/national_action_plan_for_senior_citizens.pdf

17.	Government of Nepal. Nepal Law Commission. 2018 [cited 2023 Apr 3]. The Social Security Act 2018. Available from: https://www.lawcommission.gov.np/en/wp-content/uploads/2019/07/The-Social-Security-Act-2075-2018.pdf

18.	Government of Nepal. National Planning Commission. 2024 [cited 2024 Sep 27]. The Sixteenth Plan (Fiscal Year 2024/25-2028/29). Available from: https://www.npc.gov.np/images/category/15th_plan_English_Version.pdf

19.	Government of Nepal. National Population and Housing Census 2021 (National Report) [Internet]. Kathmandu, Nepal: National Statistics Office; 2023. 614 p. Available from: https://censusnepal.cbs.gov.np/results/files/result-folder/National Report_English.pdf

20.	Cuschieri S. The STROBE guidelines. Saudi Journal of Anaesthesia. 2019;13:S31. doi:10.4103/sja.SJA_543_18

21.	Chalise HN. Provincial situation of elderly population in Nepal. American Journal of Aging Science and Research. 2020;1(1):9–11. doi:10.46439/aging.1.003

22.	Bhandari K. Process and variation of population ageing in Nepal [M.Phil. Thesis]. Tribhuvan University; 2018.

23.	Nepali S, Ghale S, Hachhethu K. Federal Nepal: Socio-cultural profiles of the seven provinces [Internet]. Kathmandu, Nepal; 2018 [cited 2023 Nov 3]. Available from: https://www.lahurnip.org/uploads/resource/file/federal-nepal-the-provinces-socio-cultural-profiles-of-the-seven-provinces.pdf

24.	Lauritsen J, Bruus M. Epidata - Comprehensive data management and basic statistical analysis system [Internet]. Odense. Denmark: The EpiData Association; 2004. Available from: http://www.epidata.dk

25.	Hayes AF, Coutts JJ. Use Omega rather than Cronbach’s alpha for estimating reliability. But…. Communication Methods and Measures. 2020 Jan 2;14(1):1–24. doi:10.1080/19312458.2020.1718629

26.	Government of Nepal. Nepal Gazette 2073 [Internet]. 2017 [cited 2023 Nov 3]. Available from: https://download.hermes.com.np/list-of-local-level-formation-of-nepal/#

27.	Bennett L, Dahal DR, Govindasamy P. Caste, ethnic and regional identity in Nepal: Further analysis of the 2006 Nepal Demographic and Health Survey [Internet]. Calverton, Maryland, USA; 2008 [cited 2023 Oct 12]. Available from: https://dhsprogram.com/pubs/pdf/FA58/FA58.pdf

28.	Government of Nepal. Nepal Gazette 2074 [Internet]. Nepal; Oct, 2017. Available from: https://www.lawcommission.gov.np/en/wp-content/uploads/2019/07/The-Act-Relating-to-Rights-of-Persons-with-Disabilities-2074-2017.pdf

29.	SAS. Cary, NC, USA: SAS Institute Inc.; 2016.

30.	Senaviratna NAMR, A. Cooray TMJ. Diagnosing multicollinearity of logistic regression model. Asian Journal of Probability and Statistics. 2019:1–9. doi:10.9734/ajpas/2019/v5i230132

31.	Chalise HN, Basnet M. Abuse of older adults residing in the community of Nepal. Journal of Gerontology & Geriatric Research. 2017;6(2). doi:10.4172/2167-7182.1000415

32.	Bhandari P, Adhikari M. Abuse experienced by elderly residing in a community of Pokhara, Nepal. Europasian Journal of Medical Sciences. 2020;2(2):77–83. doi:10.46405/ejms.v2i2.221

33.	Meurer WJ, Tolles J. Logistic regression diagnostics: Understanding how well a model predicts outcomes. JAMA. 2017;317(10):1068. doi:10.1001/jama.2016.20441

34.	Ghosh S. Deletion diagnostics in logistic regression. Journal of Applied Statistics. . 2022;1(1):1–13. doi:10.56388/as220715

35.	Dahal M, Dhakal S, Khanal S, Baral K, Mahaseth S. Linkage of depression with elder abuse among institutionalized older persons in Kathmandu valley, Nepal. Psychiatry Journal. 2021:1–8. doi:10.1155/2021/5546623

36.	Dong X. Elder abuse in Chinese populations: A global review. Journal Elder Abuse and Neglect. 2015;27(3):196–232. doi:10.1080/08946566.2015.1039154

37.	Chokkanathan S. Elder mistreatment in India. In: Handbook of aging, health and public policy. Singapore: Springer Nature Singapore; 2023. p. 1–12. doi:10.1007/978-981-16-1914-4_100-1

38.	Chalise HN. Aging in Nepal. In: Selin H, editor. Aging Across Cultures. Cham: Springer International Publishing; 2021. p. 199–210. doi:10.1007/978-3-030-76501-9_13

39.	Abolfathi Momtaz Y, Hamid TA, Ibrahim R. Theories and measures of elder abuse. Psychogeriatrics. 2013;13(3):182–8. doi:10.1111/psyg.12009

40.	Timalsina KP, Baral R, Timalsina BP, Dhungel BP, Chaulagain A. Prevalence and risk factors of elderly abuse in family environment. International Research Journal of MMC. 2023 Dec 31;4(4):36–46. doi:10.3126/irjmmc.v4i4.61928

41.

41.	Maurya P, Chattopadhyay A, Rao S, Sharma P. Understanding Elder Abuse in India: contributing Factors and Policy Suggestions. Population Ageing. 2024; 17:5-32. doi:10.1007/s12062-022-09399-x

42.	Patel AB, Mishra AJ. An empirical study of elder abuse in the state of Uttar Pradesh of India. Quaity in Ageing and Older Adults. 2018;19(2):106–16. doi:10.1108/QAOA-08-2017-0033

43.	Kaur J, Kaur J, Sujata N. Comparative study on perceived abuse and social neglect among rural and urban geriatric population. Indian Journal of Psychiatry. 2015;57(4):375. doi:10.4103/0019-5545.171852

44.	Talpur AA, Ryan T, Ali P, Hinchliff S. Elder mistreatment in South Asian communities: A review of the literature. The Journal of Adult Protection. 2018 Dec;20(5/6):193–206. doi:10.1108/JAP-06-2018-0011

45.	Bhattarai K, Adhikari AP, Gautam SP. State of urbanization in Nepal: The official definition and reality. Environmental Challenges. 2023;13:100776. doi:10.1016/j.envc.2023.100776

46.	Tareque MdI, Islam TM, Koshio A, Kawahara K. Economic well-being and elder abuse in Rajshahi district of Bangladesh. Res Aging. 2015;37(2):200–24. doi:10.1177/0164027514527833

47.	World Health Organization. Missing voices: views of older persons on elder abuse [Internet]. Geneva; 2002 [cited 2024 Apr 17]. Available from: https://iris.who.int/bitstream/handle/10665/67371/WHO_NMH_VIP_02.1.pdf
