# Supplementary material for: Prevalence of family-based elder abuse and its associated factors in Gandaki Province of Western Nepal: A cross-sectional study
Source: PLoS One. 2025 May 14;20(5):e0323713. doi: 10.1371/journal.pone.0323713 (PMC12077681; doi:10.1371/journal.pone.0323713)
Supplement: Attachment — Submitted filename: Response_to_Reviewers_auresp_2.docx [file pone.0323713.s006.docx]

**Response to Reviewers**

Dear Editor,

On behalf of all the authors, I would like to express our sincere gratitude to the Academic Editor for the time and effort dedicated to reviewing our manuscript. We have carefully addressed the editor’s comments, and a point-by-point response is included below. We believe that the revisions have undoubtedly strengthened the manuscript.

For your convenience, we have submitted two versions of manuscript: a clean version and one with track changes highlighting all the changes made in response to your feedback. Thank you in advance for your time and consideration of our revised manuscript. We look forward to your final decision.

Sincerely,

Bharat Kafle

Corresponding Author

Comments from Academic Editor

| **Feedback** | **Author response** |
| --- | --- |
| **Title Page:** Author affiliation | The address for the co-author, Aman Shrestha, has been double-checked and retained as it is, as there is no duplication. Baltimore is the name of the city, and the University of Maryland, Baltimore (UMB) is located within Baltimore City. |
| **ABSTRACT** | |
| **Background** | |
| Please use another word for ‘filial piety’ | Thank you for your suggestion. While filial piety is a common term in gerontology, I acknowledge that it may not be easily understood by readers from other disciplines. To enhance clarity, we have replaced the term with the phrase “traditional values emphasizing reverence for parents” in the abstract and defined it again where the term appears for the first time in the manuscript. |
| This is not relevant here, it suit in Methods section. Please write two sentences information about any latest data available on elder abuse of Nepal. Then end your background with your paper aims. | We appreciate your suggestion. We have made the changes accordingly. Our revised version of background reads as:  *Elder abuse is a rarely discussed public health and human rights issue in Nepal, largely due to traditional values emphasizing reverence for parents. These cultural norms can discourage recognition and reporting, especially within families. Consequently, nationwide or large-scale data on this issue is lacking. This study aims to assess the prevalence and explore the factors associated with family-based elder abuse in Gandaki province, western Nepal.* |
| **Methods** | |
| Why starting methods from sampling? Please write shortly your methods section both in abstract and details in text below. Please make your methods section stronger. You need to provide information in consistency way and take key information from main methods section | Thank you for your suggestion. We have revised the methods section for the abstract to begin with the study design, as shown below.  *A cross-sectional design was conducted interviewing 612 participants (≥ 60 years) in household settings . The study areas, representing the mountain, hill and tarai regions of Gandaki province, were randomly selected using multistage stratified sampling process. The outcome was measured using 17-item elder abuse scale. Multivariable logistic regression explored the factors associated with elder abuse.* |
| **Results** |  |
| What is the over all prevalence of abuse from your study and what is different rates of male and female, if so what causes this happened? | Thank you for your suggestion. Since the overall prevalence of elder abuse was already reported in the preceding sentence, we avoided repeating it to maintain conciseness. However, we have revised the sentence, which reads as below, to better address your concern regarding gender-based prevalence and to present the information more clearly and succinctly. We appreciate the opportunity to enhance clarity for our readers.  *The overall prevalence of elder abuse was reported at 56.4%, with caregiver neglect (50.8%) being the most prevalent sub-type. The prevalence was higher among females (66.6%) than males (42.8%).*  We have provided an explanation for the gender differences in the discussion section. However, due to the word limit, this explanation could not be included in the abstract. |
| Please present your Odds ratio results in correct way. Please follow this paper for reference  <https://journals.plos.org/plosone/article?id=10.1371/journal.pone.0283379> | Thank you for your suggestion. We have revised the acronym for odds ratio throughout the manuscript. |
| **Conclusions** | |
| Please consider key findings and policy, strength and weakness and its implication with policy recommendation. | Thank you for your suggestion regarding the conclusions section. We have revised it accordingly and provided below for your reference. However, given the scope of the abstract, we were unable to include a discussion of strengths and weaknesses, which has been covered in detail under the subheading Limitations and Strengths in the discussion section.  *The study highlights a high prevalence of elder abuse, particularly caregiver neglect, with a disproportionate impact on women. Policy recommendations include raising awareness, strengthening legal protections, and improving caregiver training to meet these challenges effectively.* |
| **Keywords** | Keywords are now listed in ascending order. |

**Introduction**

| This theory and definition need to cut down and add global, regional and national burden of elder abuse | Thank you for your suggestion. We have condensed the first paragraph to make it more concise, and it now reads as follows for your reference.  *Elder abuse is a pervasive, deeply ingrained global social issue with serious consequences for individuals and society. While terms like “mistreatment” and “abuse” are often used interchangeably, this study adopts “abuse” for consistency. In general, elder abuse refers to any intentional or negligent act that puts an older adult at risk, perpetrated by individuals who have control over, are responsible for, or have a trusted, caring relationship with the older adult [1]. Defined by the World Health Organization as harmful act within a trusted relationship [2], elder abuse can manifest in various forms, including physical, mental, sexual, or emotional, and extend to financial exploitation, acts of neglect, and abandonment, such as denying involvement in decision-making processes and restricting personal spaces [1]. Elder abuse occurs both in institutional and community settings, leading to severe health impacts and reduced quality of life [3]. Beyond individuals, elder abuse also imposes considerable financial and caregiving burdens on society and the country [4].*  The global and regional prevalence data is presented in the second paragraph, while national data is provided in the third paragraph of the introduction. We appreciate your feedback.  *Globally, a systematic review and meta-analysis, including studies from 28 countries, revealed that one in six older adults reported experiencing abuse in community settings [5], while in institutionalized settings, 64.2% of staff have admitted to abusing older adults [6]. ……………… In South Asia, cross-sectional studies indicate elder abuse prevalence rates of 14% in Bangladesh [7], between 5% to 50% in India [8] and 54% in Pakistan [9].*    *………………. While often perceived as rare in Nepali society, small-scale studies in the last ten years report a high prevalence between 46.6% to 65.6%.......................* |
| --- | --- |
| How can you say thre ecological regions with in aprovince.? Justify or replace the word | Thank you for your suggestion. We have replaced the term “ecological regions” with mountain, hill, and Tarai, and the revised sentence now reads as follows:  *To the authors’ knowledge, this study is the first to comprehensively examine elder abuse using representative samples from the mountain, hill and tarai regions within Gandaki Province, Nepal.* |
| Please end your introduction with this paer aims…  Alos reduce your introduction | Thank you for your suggestion. We have refined the study objectives accordingly, which now read as follows.  *The study aims to estimate the prevalence of elder abuse by family members and explore the factors associated with it.*  Additionally, we have significantly condensed the introduction section, reducing it to 715 words. If you have any further suggestions for streamlining it further, we would be happy to incorporate them. |

**Methods**

| This is not appropriate as this is your primary study and you do not need to write this information | Thank you for pointing this out. We have removed the statement on STROBE guidelines for reporting. |
| --- | --- |
| Write this ethical consideration information at the end of methods section | Thank you for your valuable recommendations. We have now relocated the "Ethical Considerations" subsection to the end of the Methods section, placing it after the "Data Analyses" subsection. |
| **Study design and settings:**  Please specifically write your methods section. Design means, what is this study such as crosscectional, case control etc.. Qualittaive or quantittaive etc… Please read literature about study designs and settings is something like where did you conduct study. | We appreciate your recommendation. We have revised the “Study Design and Settings” section to explicitly specify only the study design and whether a qualitative or quantitative approach was used. Additionally, we have provided information on study settings or where we conducted our study along with supporting information to justify our selection. To enhance clarity, particularly for international readers who may not be familiar with Nepal’s geographical context, we added relevant background details. However, please let us know if any of this information is unnecessary, and we will be happy to reconsider the revisions. The revised section is provided below for your reference.  *The study employed a community-based cross-sectional design, utilizing a quantitative approach for data collection and analysis. Gandaki Province (S1 Fig) was purposively selected for this study due to its high proportion of older adults, the highest among all seven provinces [23,24], its ethnic diversity with significant populations of “Brahaman/Chhetri” and “Adivasi/Janajati,” as well as a notable presence of “Dalit/Muslim” minority groups [25]. The province spans all three ecological regions of Nepal — mountains, hills, plains, or tarai — offering diverse geographical and lifestyle contexts.*  *S1 Table presents various demographic and socioeconomic indicators for Gandaki Province and its study districts, comparing them to the national average using data from the 2021 census. Briefly, Gandaki Province has a total population of 2,466,427, accounting for 8.5% of the national population [22]. Approximately 13.5% of the population in this province are 60 years or older. The old-age dependency ratio in the province is 21.25, which is higher than the national old-age dependency ratio of 16.48 [22]. Regarding development, the Human Development Index in Gandaki Province ranges from 0.48 to 0.58, closely matching the national average of 0.49 [25].* |
| Please write study population before sampling | Thank you for your suggestion. As per your suggestion, we have reorganized the content to introduce a new subheading, "Study population," before the Sampling subsection. We have added the following paragraph under this subheading to provide a clear description of the study population.  *The study population included older adults who were at least 60 years at the time of the survey. Participants were required to be permanent resident of the study area and to have proficiency in the Nepali language. Those with hearing or speech impairments that interfered with effective communication were excluded from this study.* |
| After your population information, please clearly write what is your sample size, how did you calculate and why? | Thank you for your suggestion. We revised this section accordingly. The revised paragraph is provided below for your reference.  *The sample size was calculated using one proportion formula,* $\frac{\left( Z_{1-\alpha}+Z_{1-\beta} \right)^{2} p(1-p)}{\varepsilon^{2}}$ *inbuilt in Stata/MP 13.1 [26]. The reported prevalence of elder abuse was determined at 49.1% (i.e., p=0.491) based on a previous study [11]. The power of the study was set at 80% (i.e.,* $Z_{1-\beta}$ *is 0.84) with a 95% confidence interval (*$Z_{1-\alpha}$ *is 1.96) and 10% desired margin of error (i.e.,* $\varepsilon$ *is 0.10). This initially resulted in a sample size of 194. However, after accounting for design effect of 1.5 and stratification factor of two, the sample size was recalculated to be 582. Finally, considering a 5% non-response rate, the final sample size was determined to be 613, which was rounded to 616 after sampling allocation, as illustrated in S2 Table.* |
| Before writing data collection please write data collection tools, its development process and pretesting if any or what you have done before data collection. | Thank you for your recommendation. As suggested, we have added a separate subsection titled “Survey Instruments” before the data collection subsection. In this new section, we have discussed the data collection tools, their development process, and the pretesting procedure. The revised "Survey Instruments" subsection is provided below for your reference.  *A semi-structured questionnaire was first developed in English and then translated into Nepali to facilitate data collection. To ensure accuracy and linguistic consistency, bilingual experts conducted a back translation from Nepali to English. Three co-authors in this manuscript and two local research assistants from Gandaki Province, all with prior experience in research data collection, were recruited for the fieldwork. They underwent a one-day training session that covered various aspects of the study, including its purpose, research protocols and procedures, ethical considerations, participant recruitment, interviewing techniques and safety measures. A pilot testing of the survey instrument was carried out with 30 older adults in ward no. 26 of Kathmandu Metropolitan City. While the responses from this pilot test were excluded from the primary data analysis, they proved valuable for identifying and rectifying several sensitive language issues in the questionnaire.* |
| Please clearly write data collection technique? How did you collect data and how did you keep privacy and confidentiality? | Thank you for your suggestions. We have added specific details regarding the data collection techniques as well as privacy and confidentiality measures in the data collection subsections. Below is the revised subsection on data collection for your reference:  *A paper-based questionnaire was used to interview 612 older adults through face-to-face, in-person interviews conducted between January 9, 2020, and February 26, 2020. Given the sensitive nature of the study, each interview was conducted in private space within the participant’s home to ensure confidentiality. To minimize interruptions from family members, a second research assistant was present during the interview to provide any necessary support. The collected data were subsequently digitized using data entry panels created in EpiData 3.1 [27].* |
| **Study variables** |  |
| I suggest you to define each variables with their category and what reference you have taken to category these variables? | Thank you for your valuable suggestion. We have carefully reviewed and ensured that each variable is appropriately defined. Additionally, we have cited the sources used for categorizing the variables where applicable. However, if you notice any missing references, please let us know, and we will be happy to address them. |
| Why predictors variables here? Please be aware with choosing terminology . Predictor is using first time here. Is this predictor or Explanatory variables or Exposure or Independent variable . Please be clear at first what factors you are looking for? Use same word for consistency through out the paper | Thank you for your suggestion. After considering your suggestion, we have replaced “predictors” and “outcome” with “independent variables” and “dependent variable.” We have intentionally avoided other alternative terms such as "explanatory/explained" variables to ensure clarity and uniformity. |
| It is confusing Tarai(nawarpur), Please write in methods which district belongs to which ecological zone but not in the results with in bracket. Just write study district here , not a ecological zone. But ecological zone must be define in method section and your included district, and how did you choose these with in their ecologizal zone? | Thank you for your suggestion. In the tables, we have listed only the study districts and omitted information on ecological regions to prevent any potential confusion. As per your recommendation, details regarding which district corresponds to which ecological region are now provided in the methods section under the “Sample Size and Sampling” subsection. Additionally, for further clarity, this information has been reiterated in the “Sociodemographic Factors” subsection under variable descriptions. These districts were randomly selected using a multi-stage cluster sampling approach, which is described in detail in the methods section under ‘Sample size and sampling ’ subsection. |
| Follow author guideline of PlOS one Journal | Thank you for bringing this to our attention. We have rechecked the PLOS One journal guidelines and formatted the manuscript accordingly. |
| Revisit this subheading:  Prevalence of elder abuse: Overall and sub-types | We appreciate your recommendation. We have simplified the subheading, which now reads: “Prevalence of elder abuse and its subtypes.” |
| Just keep bivariate and multivariate results in one table and result. You donot need to explain bivariate results but you must show multivariate results in the text. | Thank you for your valuable suggestion. We also appreciate your recommendation to combine the bivariate and multivariable tables into one, which is undoubtedly a valid and common approach. However, it is also standard practice to present regression analyses with adjusted and unadjusted odds ratios and 95% confidence intervals in a separate table, without including bivariate analyses.  We would like to point out that not all variables from the bivariate analyses were retained in the adjusted models. Including all variables in a combined table could result in many empty cells, particularly for variables excluded from the regression analyses (due to multicollinearity or model-building considerations using the Akaike Information Criterion). Such formatting might appear unconventional or confusing.  For these reasons, we kindly request to maintain the current format, as separating the tables allows for a clearer understanding of the relationship between variables and the model-building process. In response to your feedback, we have made the description of the bivariate table more concise. Thank you again for your thoughtful and constructive feedback. |
| You need to consider confounders in the methods section what variables you adjusted in multivariate analysis. | We appreciate your suggestion and apologize for the omission of this important information. To address this, we have added a sentence under the “Data Analyses” subsection in the methods section, clarifying the variables adjusted in the multivariate analysis. The revised sentence is provided below for your reference.  ……….Therefore, the final adjusted model (Table 4) included district, municipality type, age, gender, ethnicity, marital status, disability status, education, family type, relative visiting within the last month, healthcare visit within the last year, belief in traditional healers, reluctance to disclose health issues to family members and self-rated health. *All of these variables were treated as confounders in the regression analyses to account for their possible influence on the relationships between the predictors and outcome.* |
| Please provide reasons of similarity and differentiate of your study with comptonization studies. Please follow this rules in each discussions throughout the paper. | Thank you for your thoughtful suggestion—we truly appreciate it. The high prevalence of elder abuse in Nepal and the region is thoroughly addressed in the third paragraph, where we discuss several contributing factors, including its historical nature as a covert issue, reliance on adult children for care, financial strain, increased dependency on family caregiving, and limited governmental support. Similarly, the variations in prevalence are thoughtfully examined in the second paragraph. We have ensured a consistent format throughout the discussion wherever applicable. If you have any specific recommendations, we would be delighted to hear them and incorporate them as appropriate. Thank you again for your valuable input! |
| You need to give supplementary information heading here and keep these documents before references | Thank you for your suggestion. We have added headings for all supplementary materials after the Acknowledgments section and before the References section. Additionally, we have attached them separately as per the journal’s requirements. |
| Check each reference and follow authors guidelines. | We appreciate your recommendation. We have reviewed and made the necessary improvements to the reference section in the clean version. |
